# Machine learning approaches and genetic determinants that influence the development of type 2 diabetes mellitus: a genetic association study in Brazilian patients

**DOI:** 10.1590/1414-431X2024e13957

**Published:** 2024-12-02

**Authors:** K.F. Santos, L.P. Assunção, R.S. Santos, A.A.S. Reis

**Affiliations:** 1 Laboratório de Patologia Molecular, Instituto de Ciências Biológicas, Universidade Federal de Goiás, Goiânia, GO, Brasil; 2 Departamento de Bioquímica e Biologia Molecular, Instituto de Ciências Biológicas, Universidade Federal de Goiás, Goiânia, GO, Brasil

**Keywords:** T2DM, Genetic polymorphisms, Sex differences, Machine learning

## Abstract

This genetic association study including 120 patients with type 2 diabetes mellitus (T2DM) and 166 non-diabetic individuals aimed to investigate the association of polymorphisms in the genes *GSTM1* and *GSTT1* (gene deletion), *GSTP1* (rs1695), *ACE* (rs4646994), *ACE2* (rs2285666), *VEGF-A* (rs28357093), and *MTHFR* (rs1801133) with the development of T2DM in the population of Goiás, Brazil. Additionally, the combined effects of these polymorphisms and the possible differences between sexes in susceptibility to the disease were evaluated. Finally, machine learning models were integrated to select the main risk characteristics for the T2DM diagnosis. Risk associations were found for the *GSTT1*-null genotype in the non-stratified sample and females, and for mutant C allele of the *VEGF-A* rs28357093 polymorphism in the non-stratified sample. Furthermore, an association of heterozygous (AG) and mutant (GG) *GSTP1* genotypes was observed when combined with *GSTT1*-null. Machine learning approaches corroborated the results found. Therefore, these results suggested that *GSTT1* and *GSTP1* polymorphisms may contribute to T2DM susceptibility in a Brazilian sample.

## Introduction

Type 2 diabetes mellitus (T2DM) is a complex and progressive metabolic disease characterized by high plasma glucose levels (1). Approximately 537 million individuals worldwide are diagnosed with diabetes mellitus (DM), and it is estimated that the number of people affected by DM will rise to 643 million by 2030 and to 783 million by 2045. In Brazil, 15.7 million diabetics were registered, placing the country in 6th place in the world ranking of incidence (2).

Characterized by relative deficiency and/or insulin resistance, T2DM can cause micro- and macrovascular complications. In addition, environmental, immunological, and genetic factors contribute significantly to the pathogenesis of the disease, the clinical course, and the manifestation of complications (1). Epidemiological studies also show sex differences in risk factors, clinical manifestations, prevention, diagnosis, response to treatment, and complications of DM. Therefore, it is evident that sex differences need to be taken into account when conducting and reporting research on T2DM and in health planning, contributing to the development of specific interventions for both sexes (3).

The etiopathogenesis of T2DM is also frequently related to oxidative stress, a condition characterized by an imbalance between the production of free radicals and their correct detoxification (4). The *GSTM1* (1p13.3) (NCBI Gene ID 2944) and *GSTT1* (22q11.2) (NCBI Gene ID 2952) genes have a complete deletion polymorphism that inactivates these enzymes. The absence of these enzymes can cause a deficit in the cellular detoxification process, making tissues more susceptible to oxidative stress (5,6). In contrast, the *GSTP1* gene (11q13.2) (NCBI Gene ID 2950) has a single nucleotide polymorphism (SNP) called A313G (rs1695), characterized by the exchange of adenine for a guanine in codon 105 of exon 5 (7).

Hyperglycemia also acts as an excitatory stimulus for the renin-angiotensin system (RAS). Polymorphisms in RAS components have been evaluated in genetic association studies related to T2DM and its complications, due to the action of these enzymes in inhibiting the insulin signaling pathway and glucose metabolism, in addition to the induction of oxidative stress, causing endothelial damage, inflammation, and vascular remodeling (8).

The *ACE* gene (17q23.3) (NCBI Gene ID 1636) has an insertion/deletion (I/D) polymorphism (rs4646994) characterized by the presence (I) or absence (D) of an *Alu* segment with 287 bp in intron 16 (9), while the *ACE2* gene (Xp22.2) (NCBI Gene ID 59272) presents the SNP G8790A (rs2285666) in intron 3 (10). Studies have shown an association between this polymorphism and left ventricular hypertrophy, myocardial infarction, coronary disease, and hypertension in patients with metabolic syndrome (11-13).

Furthermore, studies have revealed increased plasma levels of vascular endothelial growth factor A (VEGF-A) in patients with DM, especially those with microvascular complications (14). In the *VEGF-A* gene (6p21.1) (NCBI Gene ID 7422), the SNP A-141C (rs28357093) stands out, characterized by the replacement of an adenine by a cytosine in the position -141 in the promoter region of the gene (15,16), which may interfere with the processes of angiogenesis, vasculogenesis, and vascular permeability.

Additionally, due to its role in cellular metabolism, the folate cycle, also known as one-carbon metabolism, has been considered an important research target on T2DM (17). This cycle includes several interconnected metabolic pathways and is responsible for homocysteine metabolism and DNA methylation (18). The *MTHFR* gene (1p36.6) (NCBI Gene ID 4524) encodes the MTHFR enzyme, responsible for the conversion of 5,10-methylenetetrahydrofolate (5,10-MTHF) into 5-methyltetrahydrofolate (5-MTHF), the form of circulating folate and the main methyl radical donor for homocysteine remethylation (19). The C677T SNP (rs1801133) in this gene causes the replacement of the amino acid alanine by valine at position 222 (20).

These underlying pathophysiological mechanisms, such as inflammation, endothelial dysfunction, and oxidative stress, are observed in the pathogenesis of T2DM. Pancreatic β cells are sensitive to free radicals, due to reduced levels of antioxidant compounds, such as glutathione peroxidase, catalase, and superoxide dismutase (21). Thus, researchers seek to understand the role of genetic polymorphisms in genes related to the oxidative stress pathway in the susceptibility and development of T2DM. Oxidative stress has been evaluated in its relationship with T2DM as a unifier of several cellular damage pathways in a hyperglycemic state (4), allowing the development of studies that evaluate several genes related to this condition.

Therefore, this study was designed to investigate the association of polymorphisms in the genes *GSTM1* (gene deletion), *GSTT1* (gene deletion), *GSTP1* (rs1695), *ACE* (rs4646994), *ACE2* (rs2285666), *VEGF-A* (rs28357093), and *MTHFR* (rs1801133) and their combined effects on the development of T2DM in the population of Goiás, Brazil. In addition, a possible difference between sexes in susceptibility to the disease was evaluated. Finally, machine learning models were integrated to select and classify the main risk characteristics for T2DM diagnosis in the sample.

## Material and Methods

### Subjects

A total of 120 patients diagnosed with T2DM treated at the Clinical Hospital of the Faculty of Medicine of the Federal University of Goiás (UFG), Brazil, were selected as the case group. The control group consisted of 166 individuals without a diagnosis of DM selected from the Clinical Analysis and Health Education Laboratory (LACES) at UFG, Goiás, Brazil. Inclusion criteria were established according to the Strengthening the Reporting of Genetic Association Studies (STREGA) guidelines for improved reporting of genetic association studies (22).

The selection criteria for the study groups were: a) Inclusion criteria: individuals aged between 30 and 90 years who underwent periodic clinical and laboratory monitoring during the data collection period; b) Exclusion criteria: patients who did not undergo laboratory monitoring, individuals who did not agree to participate in the study, and patients with DM (for the control group).

Clinical and biochemical data of the patients were collected from available medical records. Additional information on life habits, occupational history, general health conditions, previous diseases, and other anamnesis data were obtained through a questionnaire. Patients who reported smoking for more than a year before the diagnosis were considered as smokers and alcohol intake was considered for those reporting a regular intake of alcoholic beverages.

This study was conducted following the guidelines of the Research Ethics Committee (No. 1952011) of the UFG and the Ethical Principles for Medical Research Involving Human Beings of the Declaration of the World Medical Association of Helsinki. All participants provided written informed consent.

### DNA extraction and quantification

Peripheral blood samples were collected in tubes containing heparin and stored at -80°C. DNA extraction was performed using the PureLink^TM^ Genomic DNA Mini Kit (Invitrogen, USA) following the manufacturer's suggested protocol. The genomic material was evaluated and quantified with a NanoDrop™ ND-1000 spectrophotometer (ThermoFisher^®^, USA).

### Genotyping of polymorphisms in the *GSTM1*, *GSTT1*, and *ACE* genes

The genotyping of the polymorphisms in the *GSTM1*, *GSTT1*, and *ACE* genes was performed by Multiplex Real-Time Polymerase Chain Reaction (qPCR) using the fluorophore SYBR^®^ Green I (Sso Advanced™ Universal SYBR^®^ Green Supermix, Bio-Rad, USA), with discrimination of null/present and insertion/deletion genotypes by the analysis of the melting curves generated after amplification.

For the analysis of the polymorphisms in *GSTM1* and *GSTT1*, the co-amplification of the *RH92600* region, a microsatellite region used as an endogenous control of the reaction, was also performed. The primers and cycling protocols used for the amplification of *GSTM1/GSTT1* and *ACE* were previously suggested (23,24).

### Genotyping of polymorphisms in the *GSTP1*, *MTHFR*, *VEGF*-*A*, and *ACE2* genes

Genotyping of polymorphisms in the *GSTP1*, *MTHFR*, *VEGF-A*, and *ACE2* genes was performed using polymerase chain reaction-restriction fragment length polymorphism (PCR-RFLP). The PCR amplification products of the above genes were submitted to the enzymatic restriction technique using the restriction enzymes *Alw26I* (BsmAI), *Hinf I*, *HhaI*, and *Alu I*, respectively.

Subsequently, the digested fragments were visualized on an 8-15% polyacrylamide gel and stained with a 4-g/L silver nitrate solution. The primers and cycling protocols used for the above genes were reported by Harries et al. (25), Keku et al. (26), Holt et al. (15), and Benjafield et al. (27), respectively. The respective genotypes identified after enzymatic restriction are described in Table 1.

**Table 1:** Genotypes of polymorphisms in *GSTP1*, *MTHFR*, *VEGF-A*, and *ACE2* after enzymatic restriction.

Genotype	DNA bases			
	*GSTP1*	*MTHFR*	*VEGF-A*	*ACE2*
Wild	176 bp	198 bp	159 and 104 bp	466 bp
Heterozygous	176, 91, and 85 bp	198, 175, and 23 bp	159, 124, 104, and 35 bp	466, 281, and 185 bp
Mutant	91 and 85 bp	175 and 23 bp	124, 104, and 35 bp	281 and 185 bp

bp: base pairs. Genotypes A/A, C/C, A/A, and G/G correspond to the wild types of polymorphisms in the *GSTP1*, *MTHFR*, *VEGF-A*, and *ACE2* genes, respectively. Genotypes A/G, C/T, A/C, and G/A correspond to heterozygous mutants of polymorphisms in the *GSTP1*, *MTHFR*, *VEGF-A*, and *ACE2* genes, respectively. Genotypes G/G, T/T, C/C, and A/A correspond to homozygous mutants of polymorphisms in the *GSTP1*, *MTHFR*, *VEGF-A*, and *ACE2* genes, respectively.

### Statistical analysis

The statistical analysis of this study was conducted in three stages: the first stage involved a descriptive and comparative analysis of demographic and clinical variables in relation to T2DM, the second stage involved genetic models (binomial logistic regression): codominant, dominant, recessive, and overdominant, and the third approach was the application of supervised machine learning models.

In the first stage, multivariate principal component analysis (PCA) was used to evaluate the profile and interrelationship between the control and case groups. Subsequently, Student's *t*-test was used for quantitative variables, and Fisher's exact test was used for qualitative variables to assess differences and associations between demographic and clinical variables with the case and control groups. In this first approach, a significance level of 0.05 was considered.

In the second approach, the genotypes were classified as codominant, dominant, recessive, and overdominant for the application of genetic models with binary logistic regression (Equation 1). In this case, π(x) represents the probability of success given the value of the variable x (0 (control group) or 1 (case group)), X is the explanatory variable (genotypes) considered in the model, and β_0_ and β_i_ are the parameters of the logistic regression (Equation 1).

$$\log\left(\frac{\pi (\text{x})}{1-\pi (\text{x})}\right)={\text{β}}_{0}+\sum_{\text{i}=1}^{\text{n}}{\text{β}}_{\text{i}}{\text{X}}_{\text{i}}$$ (Eq. 1)

Subsequently, using the estimated parameters, the odds ratio (OR) (Equation 2) and the confidence intervals (CI) with a confidence level of 95% (Equation 3) were calculated:

$$\text{OR}=\frac{\pi (1)\{1-\pi (0)\}}{\pi (0)\{1-\pi (1)\}}=e^{\beta }$$ (Eq. 2)

$${\text{and}\;(\text{OR}}_{\text{LL}}{;\;\text{OR}}_{\text{UL}})\;=\;\text{exp}(\hat{\text{β}}\pm Z_{\left(1-\alpha /2\right)}{\text{S}}_{\hat{\text{β}}})$$ (Eq. 3)

in which, OR_LL_ is the lower limit of the OR, OR_UL_ is the upper limit of the OR, Z is the value of the probability distribution, α is the significance level, and ${\text{S}}_{\hat{\text{β}}}$ is the standard deviation of the parameter $\hat{\text{β}}$

Furthermore, for the diagnosis model, the Akaike Information Criterion (AIC) and the Bayesian Information Criterion (BIC) were used according to Burnham and Anderson (28). Subsequently, the data were stratified by sex and the genetic models were created using logistic regression.

Additionally, to perform an initial screening of the interactions between all the SNPs evaluated in the study with T2DM, the chi-squared test was applied with a significance level of 0.10. Consecutively, all SNPs with a P-value less than 0.10 were subjected to a binary logistic regression analysis with a significance level of 0.05, since this approach has greater statistical power compared to the chi-squared association test. Therefore, the results were interpreted based on the logistic regression analysis.

In the third stage, the data were modeled with supervised machine learning to identify variables that helped in the diagnosis of T2DM. Initially, the database was randomly split into training (70.00%) and testing (30.00%) sets, and then the training data were used for machine learning, using the following models: logistic regression (LR), classification and regression tree (CART), K-nearest neighbors (KNN), support vector machine (SVM), and random forest (RF) (29-32). Additionally, to avoid possible sample selection biases in the training set, cross-validation was performed. To verify the importance of each covariate in the database for the models, the permutation test with accuracy score was used. Subsequently, the diagnosis of the supervised models was carried out in two complementary ways: by calculating accuracy, precision, and recall from the confusion matrix and by estimating the area under the curve (AUC) of the receiver operating characteristic (ROC) curve. Thus, for selecting the best models, accuracy, precision, recall, and AUC values of the ROC curve close to 1 were evaluated.

All analyses were performed with the aid of spreadsheets, using R software version 4.0.2 and Python version 3.12.2.

## Results

### Clinical data

A total of 120 T2DM patients and 166 individuals without DM were evaluated considering demographic, clinical, and laboratory characteristics (Tables 2 and 3). The mean age of the case and control groups was 60.47±9.87 and 57.89±9.91, respectively, with a significant difference between groups (P=0.03). Both groups were predominantly composed of women, however, there was no statistical difference for this variable (P=0.06) (Table 2).

**Table 2:** Demographic and clinical characterization of diabetic (case) and non-diabetic (control) groups.

Variable	Control	Case	P
Age (mean±SD)	57.89±9.91	60.47±9.87	0.03^a*^
Gender, n (%)			
Female	96 (57.83)	83 (69.17)	0.06^b^
Male	70 (42.17)	37 (30.83)	
Alcohol intake, n (%)			
No	104 (62.65)	91 (75.83)	0.77^b^
Yes	43 (25.90)	29 (24.17)	
Not described	19 (11.45)	0 (0.00)	
Smoking, n (%)			
No	90 (54.22)	63 (52.50)	0.17^b^
Yes	57 (34.34)	57 (47.50)	
Not described	19 (11.44)	0 (0.00)	

*P<0.05. ^a^Student's *t*-test; ^b^Fisher's exact test.

Furthermore, alcohol intake and smoking showed no statistical difference between groups (P=0.77 and P=0.17, respectively) (Table 2). Statistically significant differences (P<0.05) between the groups were found for fasting glycemia, cholesterol, triglycerides, high-density lipoprotein cholesterol (HDL-C), low-density lipoprotein cholesterol (LDL-C), very low-density lipoprotein cholesterol (VLDL-C), body mass index (BMI), and blood pressure (Table 3).

**Table 3:** Comparison of clinical parameters between diabetic (case) and non-diabetic (control) groups.

Variable	Control	Case	P
Fasting glycemia (mg/dL)	86.92±10.45	186.01±78.08	2.00E-16*
Cholesterol (mg/dL)	156.91±30.02	183.81±44.15	2.94E-08*
Triglycerides (mg/dL)	122.30±41.34	156.80±91.29	0.000*
HDL-C (mg/dL)	49.09±10.38	46.15±12.93	0.041*
LDL-C (mg/dL)	83.40±29.10	110.39±34.84	4.60E-11*
VLDL-C (mg/dL)	24.46±8.27	31.36±18.25	0.000*
Systolic pressure (mmHg)	123.28±10.46	136.64±18.86	5.16E-11*
Diastolic pressure (mmHg)	75.82±7.81	83.30±10.95	1.12E-09*
BMI	27.00±5.44	28.80±5.54	0.006*
Creatinine	0.98±0.20	0.96±0.31	0.427

Data are reported as mean and SD. *P<0.05. Student's *t*-test. BMI: body mass index; HDL-C: high-density lipoprotein cholesterol; LDL-C: low-density lipoprotein cholesterol; VLDL-C: very low-density lipoprotein cholesterol.

### Genetic analysis

**Figure 1:**
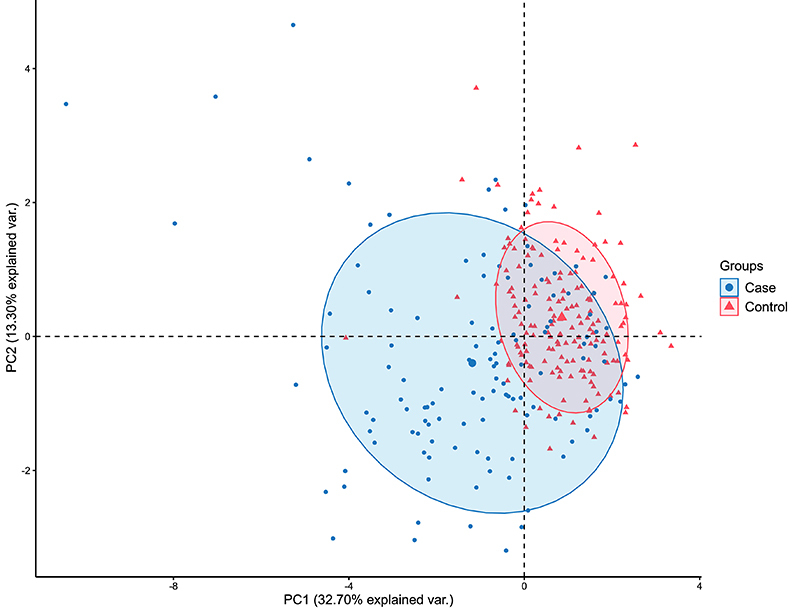
Principal component analysis (PCA) of case and control groups.

The PCA analysis revealed that component 1 (PC1) explains 32.70% of the data variability, while component 2 (PC2) explains 13.30%, demonstrating that components 1 and 2 explain 46.00% of the variability of the data set. Furthermore, the case and control groups did not present a relevant contrast, since the limits of the clusters of each group overlapped. Thus, this small variation between groups allowed using PCA as a covariate and resizing the data (Figure 1). However, there was greater variability in the group of patients with T2DM when compared to the control group, that is, the control group (without T2DM) presented greater homogeneity in clinical and demographic data compared to the case group (Figure 1). Therefore, this heterogeneity in the case group may be related to T2DM, as in individual comparisons between groups, statistical differences were observed for fasting glycemia, cholesterol, triglycerides, HDL-C, LDL-C, VLDL-C, systolic pressure, diastolic pressure, BMI, and creatine (Table 3).

The identification of polymorphisms in the *GSTM1*, *GSTT1*, and *ACE* genes by qPCR and *GSTP1*, *MTHFR*, *VEGF-A*, and *ACE2* by PCR-RFLP are detailed in Supplementary Figures S1 to S6. The genotype and allele frequencies for the different models of inheritance in the case and control groups are presented in Table 4. There was a 3.16-fold increased risk for the *GSTT1*-null genotype and the development of T2DM (P=0.000267), and a significant difference of the mutant C allele of the *VEGF-A* rs28357093 between groups (P=0.048).

**Table 4:** Genotypic and allele frequencies of the investigated polymorphisms and the association between diabetic (case) and non-diabetic (control) groups, using models of inheritance.

	Genotypic frequency					
Gene	Model	Genotype	Control, n (%)	Case, n (%)	OR (95%CI)	P
*GSTT1*	Dominant	P	146 (88.48)	85 (70.83)	Ref	–
		N	19 (11.52)	35 (29.17)	3.164 (1.721–5.972)	0.000267*
*GSTM1*	Dominant	P	95 (57.57)	70 (58.33)	Ref	–
		N	70 (42.43)	50 (41.67)	0.969 (0.600–1.560)	0.898
*GSTP1*	Codominant	AA	38 (36.54)	56 (47.86)	Ref	–
		AG	56 (53.85)	44 (37.60)	0.533 (0.299–0.940)	0.030*
		GG	10 (9.61)	17 (14.54)	1.153 (0.482–2.867)	0.751
	Dominant	AA	38 (36.54)	56 (52.14)	Ref	–
		AG + GG	66 (63.46)	61 (47.86)	0.627 (0.364–1.072)	0.091
	Recessive	AA + AG	94 (90.38)	100 (85.47)	Ref	–
		GG	10 (9.62)	17 (14.53)	1.598 (0.707–3.786)	0.269
	Overdominant	AA + GG	48 (46.15)	73 (62.39)	Ref	–
		AG	56 (53.85)	44 (37.61)	0.516 (0.300–0.881)	0.016*
*ACE*	Codominant	II	37 (25.87)	34 (28.33)	Ref	–
		ID	79 (55.24)	62 (51.66)	0.854 (0.481–1.515)	0.589
		DD	27 (18.89)	24 (20.01)	0.967 (0.469–1.990)	0.928
	Dominant	II	37 (25.88)	34 (28.34)	Ref	–
		ID + DD	106 (74.12)	86 (71.66)	0.882 (0.511–1.526)	0.655
	Recessive	II + ID	116 (81.12)	96 (80.00)	Ref	–
		DD	27 (18.88)	24 (20.00)	1.074 (0.579–1.982)	0.819
	Overdominant	II + DD	64 (44.75)	58 (48.33)	Ref	–
		ID	79 (55.25)	62 (51.67)	0.865 (0.531–1.409)	0.562
*ACE2*	Codominant	GG	85 (58.62)	72 (60.00)	Ref	–
		GA	42 (28.96)	42 (35.00)	1.180 (0.694–2.009)	0.540
		AA	18 (12.42)	6 (5.00)	0.393 (0.136–0.994)	0.061
	Dominant	GG	85 (58.62)	72 (60.00)	Ref	–
		GA + AA	60 (41.38)	48 (40.00)	0.944 (0.576–1.545)	0.82
	Recessive	GG + GA	127 (87.59)	114 (95.00)	Ref	–
		AA	18 (12.41)	6 (5.00)	0.371 (0.130–0.919)	0.042*
	Overdominant	GG + AA	103 (71.03)	78 (65.00)	Ref	–
		GA	42 (28.97)	42 (35.00)	1.320 (0.785–2.222)	0.294
*MTHFR*	Codominant	CC	74 (51.03)	66 (55.46)	Ref	–
		CT	67 (46.21)	49 (41.17)	0.819 (0.498–1.345)	0.433
		TT	4 (2.76)	4 (3.37)	1.121 (0.255–4.911)	0.875
	Dominant	CC	74 (51.03)	66 (55.46)	Ref	–
		CT + TT	71 (48.97)	53 (44.54)	0.836 (0.513–1.360)	0.473
	Recessive	CC + CT	141 (97.24)	115 (96.64)	Ref	–
		TT	4 (2.76)	4 (3.36)	1.226 (0.284–5.28)	0.777
	Overdominant	CC + TT	78 (53.79)	70 (58.82)	Ref	–
		CT	67 (46.21)	49 (41.18)	0.814 (0.498–1.328)	0.413
*VEGF‐A*	Codominant	AA	136 (93.79)	102 (87.18)	Ref	–
		AC	8 (5.52)	14 (11.96)	2.333 (0.962–6.032)	0.066
		CC	1 (0.69)	1 (0.86)	1.333 (0.052–33.985)	0.839
	Dominant	AA	136 (93.79)	102 (87.18)	Ref	–
		AC + CC	9 (6.21)	15 (12.82)	2.222 (0.950–5.480)	0.070
	Recessive	AA + AC	144 (99.31)	116 (99.14)	Ref	–
		CC	1 (0.69)	1 (0.86)	1.241 (0.048–31.618)	0.878
	Overdominant	AA + CC	137 (94.48)	103 (88.03)	Ref	–
		AC	8 (5.52)	14 (11.97)	2.327 (0.959–6.022)	0.067

	Allele frequency					
Gene	Model	Alleles	Control, n (%)	Case, n (%)	OR (95%CI)	P
*GSTP1*	–	A	132 (63.46)	156 (66.66)	–	0.485
		G	76 (36.54)	78 (33.34)		
*ACE*	–	I	153 (53.49)	130 (54.16)	–	0.930
		D	133 (46.51)	110 (45.84)		
*ACE2*	–	G	212 (73.10)	186 (77.50)	–	0.267
		A	78 (26.90)	54 (22.50)		
*MTHFR*	–	C	215 (74.14)	181 (76.05)	–	0.683
		T	75 (25.86)	57 (23.95)		
*VEGF‐A*	–	A	280 (96.55)	218 (93.16)	–	0.048*
		C	10 (3.45)	16 (6.84)		

Logistic regression. *P<0.05. A: adenine; C: cytosine; D: deletion; G: guanine; I: insertion; N: Null; OR: odds ratio; P: present; Ref: reference; T: thymine.

Moreover, a reduced risk of disease development was observed with the presence of the AG genotype of the *GSTP1* rs1695 polymorphism in the codominant (OR=0.533, CI=0.299-0.940, P=0.030) and overdominant models (OR=0.516, CI=0.300-0.881, P=0.016), and AA of the *ACE2* rs2285666 in the recessive model (OR=0.371, CI=0.130-0.919, P=0.042).

Supplementary Table S1 shows the genotype and allele frequencies for the different models of inheritance in both groups stratified by sex. Women with the *GSTT1*-null genotype demonstrated a 3.66-fold increased risk of developing T2DM (P=0.001). Additionally, a reduced risk of developing the disease in women was observed with the presence of the AG genotype of the *GSTP1* rs1695 polymorphism in the codominant (OR=0.473, CI=0.23-0.94, P=0.035) and overdominant models (OR=0.479, CI=0.24-0.91, P=0.027).

The selection of polymorphisms in different genes for combined analysis is shown in Supplementary Table S2. Polymorphisms in *GSTP1*, *ACE2*, and *GSTT1* in the codominant model, *GSTP1* and *GSTT1* in the dominant and overdominant models, and polymorphisms in *ACE2* and *GSTT1* in the recessive model were selected.

From screening, the analysis of the genotypic combinations was performed with the codominant, dominant, recessive, and overdominant models of inheritance (Table 5). In the codominant model, the combination of *GSTP1*-wild (AA) and *GSTT1*-null genotypes revealed a significant association with the development of T2DM (OR=3.70, CI=1.23-13.83, P=0.02). A risk association was also found for the *ACE2*-wild (GG) and *GSTT1*-null genotypic combination (OR=4.48, CI=1.67-14.30, P=0.00) (Table 5).

**Table 5:** Frequency distribution of genotypic combinations and risk analysis in diabetic and non-diabetic groups according to inheritance models.

Codominant model	Control	Case	OR (95%CI)	P
*GSTP1/GSTT1*				
W/p	34	39	Ref	Ref
W/n	4	17	3.70 (1.23-13.83)	0.02
H/p	50	31	0.54 (0.28-1.02)	0.06
H/n	6	12	1.74 (0.60-5.47)	0.31
M/p	7	11	1.36 (0.48-4.09)	0.55
M/n	3	5	1.45 (0.33-7.50)	0.62
*GSTP1/ACE2*				
W/W	22	30	Ref	Ref
W/H	11	24	1.60 (0.65-4.03)	0.30
W/M	5	2	0.29 (0.03-1.49)	0.16
H/W	31	28	0.66 (0.31-1.39)	0.28
H/H	19	13	0.24 (0.03-1.17)	0.10
H/M	6	2	0.50 (0.20-1.21)	0.13
M/W	7	11	1.15 (0.38 -3.57)	0.80
M/H	3	5	1.22 (0.27-6.46)	0.79
M/M	0	0	-	-
*ACE2/GSTT1*				
W/p	55	49	Ref	Ref
W/n	5	20	4.48 (1.67-14.30)	0.00
H/p	25	29	1.30 (0.67-2.52)	0.43
H/n	8	13	1.82 (0.70-4.95)	0.22
M/p	11	3	0.30 (0.06-1.04)	0.08
M/n	0	1	2.37 E+06 (1.72E-72-inf)	0.98
*GSTP1/ACE2/GSTT1*				
W/W/p	19	20	Ref	Ref
W/W/n	3	10	3.16 (0.82-15.76)	0.11
W/H/p	10	17	1.61 (0.59-4.49)	0.52
W/H/n	1	7	6.65 (1.04-130.51)	0.08
W/M/p	5	2	0.38 (0.05-1.99)	0.28
W/M/n	0	0	-	-
H/W/p	31	21	0.64 (0.27 -1.48)	0.30
H/W/n	0	7	1.48E+07 (9.78E-29-inf)	0.98
H/H/p	13	9	0.65 (0.22-1.87)	0.43
H/H/n	6	4	0.63 (0.14-2.56)	0.52
H/M/p	6	1	0.15 (0.00-1.04)	0.10
H/M/n	0	1	1.48E+07 (7.29E-206-inf)	0.99
M/W/p	5	8	1.52 (0.42-5.81)	0.52
M/W/n	2	3	1.42 (0.21-11.73)	0.71
M/H/p	2	3	1.42 (0.21-11.73)	0.71
M/H/n	1	2	1.90 (0.16-42.88)	0.61
M/M/p	0	0	-	-
M/M/n	0	0	-	-

Dominant model	Control	Case	OR (95%CI)	P
*GSTP1/GSTT1*				
W/p	57	42	Ref	Ref
W/n	9	17	2.56 (1.06–6.54)	0.04
H+M/p	34	39	1.55 (0.84–2.87)	0.15
H+M/n	4	17	5.76 (1.96–21.17)	0.00

Recessive model	Control	Case	OR (95%CI)	P
*ACE2/GSTT1*				
H+M/n	11	3	Ref	Ref
H+M/p	0	1	7.76E+06 (7.35E–72‐inf)	0.98
W/n	80	78	3.57 (1.06–16.24)	0.05
W/p	13	33	9.30 (2.45–46.34)	0.00

Overdominant model	Control	Case	OR (95%CI)	P
*GSTP1/GSTT1*				
W+M/p	50	31	Ref	Ref
W+M/n	6	12	3.22 (1.13–10.09)	0.03
H/p	41	50	1.96 (1.07–3.64)	0.02
H/n	7	22	5.06 (2.01–14.11)	0.00

Logistic regression. W: Wild; H: Heterozygous; M: Mutant; p: present; n: null; Ref: reference.

In the dominant model, risk associations were demonstrated in the genotypic combinations *GSTP1*-wild (AA) and *GSTT1*-null (OR=2.56, CI=1.06-6.54, P=0.04) and *GSTP1*-heterozygous+mutant (AG+GG) and *GSTT1*-null (OR=5.76, CI=1.96-21.17, P=0.00). In the recessive model, the genotypic combination of *ACE2*-wild (GG) with *GSTT1*-null (OR=3.57, CI=1.06-16.24, P=0.05) or present (OR=9.30, CI=2.45-46.34, P=0.00) demonstrated an association with the development of T2DM (Table 5).

The overdominant model showed association in all combinations performed: *GSTP1*-wild+mutant (AA+GG) and *GSTT1*-null (OR=3.22, CI=1.13-10.09, P=0.03), *GSTP1*-heterozygous (AG) and *GSTT1*-present (OR=1.96, CI=1.07-3.64, P=0.02), and *GSTP1*-heterozygous (AG) and *GSTT1*-null (OR=5.06, CI=2.01-14.11, P=0.00) (Table 5).

### Machine learning approaches

Seeking an approach to predicting T2DM based on explanatory variables, five machine learning models were tested (LR, CART, KNN, SVM, and RF). There are few studies on the application of machine learning in the biological and medical areas, and the available work shows great divergence in the models used. Therefore, we used the top five supervised machine learning models to screen the best predictive models, a common approach for machine learning model selection.

**Figure 2:**
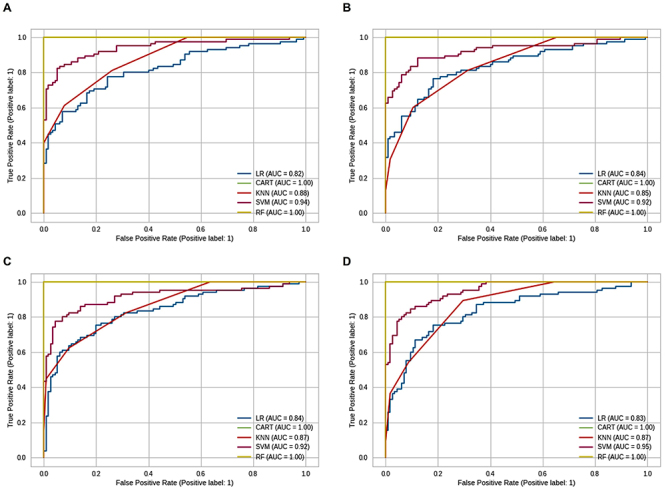
Receiver operating characteristic (ROC) curve plots of the ability to distinguish classes. **A**, codominant model of inheritance; **B**, dominant model of inheritance; **C**, recessive model of inheritance; **D**, overdominant model of inheritance. LR: logistic regression; CART: classification and regression tree; KNN: K-nearest neighbors; SVM: support vector machine; RF: random forest.

The selection of the best model was based on the accuracy, precision, recall, and AUC parameters of the ROC curve. The AUC of the ROC curve revealed the ability of the models to distinguish classes (case and control). Models with 100% wrong predictions have an AUC=0, while models with 100% correct predictions have an AUC=1. In this way, we confirmed that the CART and RF models had the best fit to the training data in all inheritance models (Figure 2).

**Figure 3:**
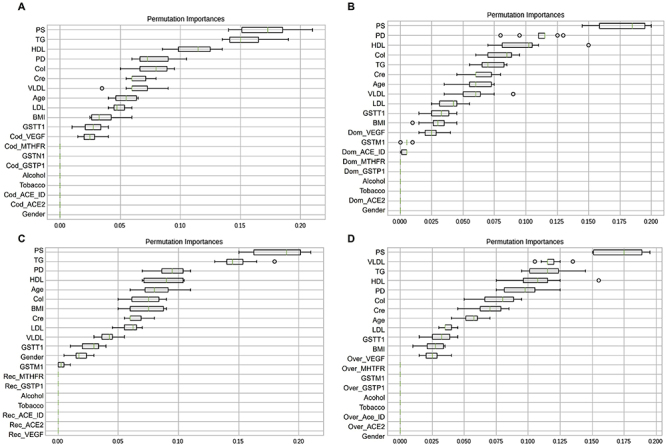
Permutation test with the classification and regression tree (CART) model for each inheritance model. **A**, codominant; **B**, dominant; **C**, recessive; **D**, overdominant. *ACE*: angiotensin converting enzyme; BMI: body mass index; Cod: codominant; Col: cholesterol; Cre: creatinine; Dom: dominant; *GSTM1*: glutathione S-transferase mu 1; *GSTP1*: glutathione S-transferase pi 1; *GSTT1*: glutathione S-transferase theta 1; HDL: high density lipoproteins; ID: insertion/deletion; LDL: low density lipoprotein; *MTHFR*: methylenetetrahydrofolate reductase; Over: overdominant; PD: diastolic pressure; PS: systolic pressure; Rec: recessive; TG: triglycerides; *VEGF*: vascular endothelial growth factor; VLDL: very low-density lipoprotein.

**Figure 4:**
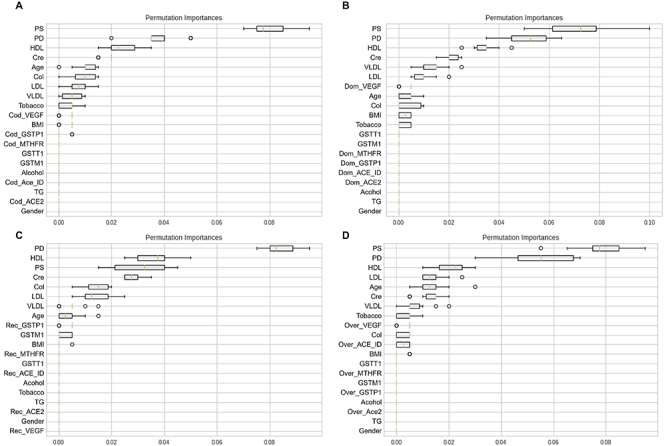
Permutation test with the random forest (RF) model for each inheritance model. **A**, codominant; **B**, dominant; **C**, recessive; **D**, overdominant. *ACE*: angiotensin converting enzyme; BMI: body mass index; Cod: codominant; Col: cholesterol; Cre: Creatinine; Dom: dominant; *GSTM1*: glutathione S-transferase mu 1; *GSTP1*: glutathione S-transferase pi 1; *GSTT1*: glutathione S-transferase theta 1; HDL: high density lipoproteins; ID: insertion/deletion; LDL: low density lipoprotein; *MTHFR*: methylenetetrahydrofolate reductase; Over: overdominant; PD: diastolic pressure; PS: systolic pressure; Rec: recessive; TG: triglycerides; *VEGF*: vascular endothelial growth factor; VLDL: very low-density lipoprotein.

The training phase revealed the importance of each explanatory variable within the models used (LR, CART, KNN, SVM, and RF) (Figures 3 and 4, and Supplementary Figures S7 to S9); the fasting glycemia variable was excluded to avoid this confounding bias. Table 6 shows the performance of the models based on accuracy, precision, and recall in the training data. Values closer to 1 indicate the most appropriate model.

**Table 6:** Performance evaluation of machine learning models based on accuracy, precision, and recall in the training data for each inheritance model.

Model	Accuracy	Precision	Recall
Codominant			
LR	0.76	0.75	1.00
CART	1.00	1.00	1.00
KNN	0.79	0.76	0.92
SVM	0.87	0.83	0.96
RF	1.00	1.00	1.00
Dominant			
LR	0.77	0.77	1.00
CART	1.00	1.00	1.00
KNN	0.77	0.75	0.89
SVM	0.85	0.81	0.95
RF	1.00	1.00	1.00
Recessive			
LR	0.78	0.78	1.00
CART	1.00	1.00	1.00
KNN	0.78	0.76	0.90
SVM	0.87	0.85	0.93
RF	1.00	1.00	1.00
Overdominant			
LR	0.78	0.77	1.00
CART	1.00	1.00	1.00
KNN	0.75	0.72	0.91
SVM	0.86	0.83	0.95
RF	1.00	1.00	1.00

LR: logistic regression; CART: classification and regression tree; KNN: K-nearest neighbors; SVM: support vector machine; RF: random forest.

Therefore, the CART and RF models showed the best performance in the training data in all inheritance models (Table 6). However, caution is needed with well-adjusted models, since they can be great for adjusting data, but inefficient in predicting values (overfitting). Figures 3 and 4 show the permutation test performed for each inheritance model with the CART and RF models. The y-axis of the graphs shows the variables used and the x-axis measures the importance of each variable in the model.

In the CART model, the main variables found were blood pressure, HDL-C, and triglycerides in the codominant inheritance model; blood pressure, HDL-C, and cholesterol in the dominant inheritance model; blood pressure, triglycerides, and HDL-C in the recessive inheritance model; and systolic pressure, VLDL-C, triglycerides, and HDL-C in the overdominant inheritance model (Figure 3). While in the RF model, the main variables identified were blood pressure, HDL-C, and creatinine in all inheritance models (Figure 4).

In the testing phase, each model was validated with the accuracy, precision, and recall parameters in each inheritance model (Table 7). In the accuracy parameter, the CART and RF models were the most adequate in all inheritance models, in terms of accuracy, the LR and RF models received the best scores, and in the recall parameter, the SVM, KNN, and RF models had the best fit to the data. The RF model had the best fit to the data set in general, showing higher values in all evaluated parameters.

**Table 7:** Performance evaluation of machine learning models based on accuracy, precision, and recall in the test data for each inheritance model.

Model	Accuracy	Precision	Recall
Codominant			
LR	0.76	0.84	0.86
CART	1.00	0.79	0.82
KNN	0.79	0.73	0.80
SVM	0.87	0.78	0.94
RF	1.00	0.85	0.92
Dominant			
LR	0.77	0.87	0.82
CART	1.00	0.83	0.78
KNN	0.77	0.71	0.84
SVM	0.85	0.78	0.88
RF	1.00	0.85	0.94
Recessive			
LR	0.78	0.86	0.84
CART	1.00	0.79	0.74
KNN	0.78	0.73	0.86
SVM	0.87	0.75	0.86
RF	1.00	0.84	0.84
Overdominant			
LR	0.78	0.82	0.80
CART	1.00	0.84	0.82
KNN	0.75	0.71	0.84
SVM	0.86	0.75	0.84
RF	1.00	0.82	0.94

LR: logistic regression; CART: classification and regression tree; KNN: K-nearest neighbors; SVM: support vector machine; RF: random forest.

## Discussion

T2DM is a polygenic and multifactorial disease diagnosed in 90-95% of diabetics, being considered the most common form of diabetes in individuals over 45 years of age (1). Analyses revealed significant differences between groups for age, fasting glycemia, cholesterol, triglycerides, HDL-C, LDL-C, VLDL-C, BMI, and blood pressure (Tables 2 and 3).

These results agreed with scientific reports, which consider advanced age, obesity, sedentary lifestyle, and presence of metabolic syndrome components, such as arterial hypertension and dyslipidemia, as main risk factors for the development of T2DM (1). Thus, the differences found may reflect the degree of metabolic decompensation observed in T2DM patients (4).

In this study, genetic analysis revealed that the *GSTT1*-null genotype is associated with a statistically significant 3.16-fold higher risk of T2DM (Table 4). The combined analysis of the genotypes (Table 5) corroborated the previously found associations. The *GSTT1*-null genotype was associated with disease development in all inheritance models. Additionally, the risk association found in the genotypic combinations *GSTT1*-null + *GSTP1*-heterozygote+mutant (AG+GG) in the dominant model, and *GSTT1*-null + *GSTP1*-heterozygote (AG) in the overdominant model was highlighted. Previous studies have also found an association of *GSTT1* and *GSTP1* polymorphisms with the development of T2DM both individually and in combination (33-35).

Glutathione S-transferases (GSTs) conjugate reduced glutathione with reactive compounds. Consequently, these compounds become more hydrophilic and are excreted. Probably due to the action of GSTs in cellular detoxification, individuals with deletion of these genes may have reduced antioxidant defenses. The enzymes of this family also act in other intracellular mechanisms, such as cell replication, modulation of signaling pathways, apoptosis, and drug resistance (5). Meta-analyses report the *GSTT1* polymorphism as a risk factor for T2DM and its complications (33,34). Furthermore, the presence of this gene is reported to be a protective factor against the development of the disease (21).

A significant difference was also found for the mutant C allele of *VEGF-A* rs28357093 polymorphism (Table 4). This is the first study to evaluate this SNP in the pathogenesis of T2DM and there are no studies evaluating the effect of the SNP on gene expression and protein synthesis. However, according to the literature, the control of VEGF-A expression is essential for the maintenance of pancreatic islet vessels and for glucose homeostasis (36).

VEGF-A signaling from β cells to endothelial cells maintains the endocrine vasculature, while the opposite signaling acts on pancreatic development, insulin secretion, β cell proliferation, and adequacy of cell mass to metabolic changes (36). Studies have reported increased levels of VEGF-A in T2DM patients and a risk association of this gene with the development of microvascular complications (14).

Additionally, the analysis demonstrated a protective association of the AG genotypes of the *GSTP1* rs1695 polymorphism in the codominant and overdominant models and AA of the *ACE2* rs2285666 in the recessive model (Table 4). In contrast to this study, heterozygous (AG) genotypes of the rs1695 polymorphism in the *GSTP1* gene (35) and mutant (AA) of the *ACE2* rs2285666 polymorphism (37) were related to higher risk of developing T2DM. There were no significant associations with other analyzed variants.

Genetic analyzes were also performed with the sample stratified by sex. Results showed an increased risk in women with the *GSTT1*-null genotype, and a decreased risk with the AG genotype of the *GSTP1 rs1695* polymorphism in the codominant and overdominant models of inheritance (Supplementary Table S1), in agreement with the associations found in the analyses with both sexes. We evaluated 83 women in the case group and 96 in the control group, so caution should be taken when interpreting the results due to the small sample and the higher frequency of women in both groups compared to men.

Rao et al. (38) reported that the combination of *GSTM1*-null and *GSTT1*-null genotypes confer a higher risk in women than in men of developing T2DM, and that diabetic woman had lower levels of GST activity. Associations between insulin resistance biomarkers, metabolic syndrome, inflammation, and endothelial dysfunction were also described more in women, since diabetic women are subject to greater changes in coagulation, inflammation, and vascular function (3).

Although medical interest in these specific differences between sexes is increasing, the underlying mechanisms that influence this differentiation are not fully elucidated. Therefore, the need to include a perspective of differences between sexes in conducting and reporting research on T2DM and in health planning is evident, helping in the elaboration of specific interventions for men and women (1,3).

Polymorphisms in genes of the GST family affect antioxidant defenses, favoring the onset of oxidative stress with increased production of free radicals (39). Therefore, these genetic variants seem promising for predicting the development of T2DM, and additional studies are needed to elucidate the molecular mechanisms underlying these polymorphisms and evaluate their interactions in T2DM and disease complications.

Additionally, clinical and genetic data were used to apply supervised machine learning, which consists of algorithms that allow the computer to learn from the available data. From the cross-validation (training and testing stage) the machine's performance is evaluated, measuring its effectiveness in predicting results based on the rules it learned in the training stage. These models are a powerful alternative to conventional statistical tests, providing greater flexibility in describing complex data (40). In this study, the method was used to determine which genetic and/or environmental variables lead to increased susceptibility of an individual to T2DM.

The performance evaluation revealed that the CART and RF models were the most adjusted for the training data set, and the RF model for the test data set (Tables 6 and 7). In the CART model, the variables blood pressure, HDL-C, VLDL-C, triglycerides, and cholesterol were the most relevant for predicting the phenotype. In the RF model, the variables blood pressure, HDL-C, LDL-C, and creatinine stood out. Thus, supervised machine learning approaches corroborate the clinical data found in this study.

The use of machine learning methods to predict diagnoses is increasing due to their applicability to different areas of health care. However, as this is a new approach, there is a lack of studies on the application of these models to predict human diseases, highlighting the importance of new reproducible studies in the general population to optimize the methods to predict the outcome in different populations.

Therefore, understanding the genetic factors associated with the development of T2DM in the Brazilian population can elucidate the role of molecular mechanisms in the susceptibility to the disease. The importance of these studies in Brazil is also highlighted for building knowledge about Brazilian genetics, which is greatly influenced by the mixture of ancestries not found in other populations. Furthermore, this study sought to contribute to the identification of possible biomarkers of susceptibility to T2DM, which indicated possible prognostic criteria and specific health interventions for both sexes.

## Supplementary Material

Click here to view [pdf].

## References

[1] American Diabetes Association (2018). Classification and Diagnosis of Diabetes: Standards of Medical Care in Diabetes-2018. Diabetes Care.

[2] International Diabetes Federation (IDF) (2021). IDF Diabetes Atlas.

[3] Day S, Wu W, Mason R, Rochon PA (2019). Measuring the data gap: inclusion of sex and gender reporting in diabetes research. Res Integr Peer Rev.

[4] Yaribeygi H, Sathyapalan T, Atkin SL, Sahebkar A (2020). Molecular mechanisms linking oxidative stress and Diabetes Mellitus. Oxid Med Cell Longev.

[5] Allocati N, Masulli M, Di Ilio C, Federici L (2018). Glutathione transferases: substrates, inhibitors and pro-drugs in cancer and neurodegenerative diseases. Oncogenesis.

[6] Suthar PC, Purkait P, Uttaravalli K, Sarkar BN, Ameta R, Sikdar M (2018). Glutathione S-transferase M1 and T1 null genotype frequency distribution among four tribal populations of western India. J Genet.

[7] Wang M, Li Y, Lin L, Song G, Deng T (2016). GSTM1 null genotype and GSTP1 ILE105Val polymorphism are associated with alzheimer's disease: a meta-analysis. Mol Neurobiol.

[8] Hsiao CF, Sheu WWH, Hung YJ, Lin MW, Curb D, Ranadex K (2012). The effects of the renin-angiotensin-aldosterone system gene polymorphisms on insulin resistance in hypertensive families. J Renin-Angiotensin-Aldosterone Syst.

[9] Rigat B, Hubert C, Alhenc-Gelas F, Cambien F, Corvol P, Soubrier F (1990). An insertion/deletion polymorphism in the angiotensin I-converting enzyme gene accounting for half the variance of serum enzyme levels. J Clin Invest.

[10] Rahimi Z (2016). The role of renin angiotensin aldosterone system genes in diabetic nephropathy. Can J Diabetes.

[11] Lieb W, Graf J, Götz A, König IR, Mayer B, Fischer M (2006). Association of angiotensin-converting enzyme 2 (ACE2) gene polymorphisms with parameters of left ventricular hypertrophy in men: results of the MONICA Augsburg echocardiographic substudy. J Mol Med (Berl).

[B12] Yang W, Huang W, Su S, Li B, Zhao W, Chen S (2006). Association study of ACE2 (angiotensin I-converting enzyme 2) gene polymorphisms with coronary heart disease and myocardial infarction in a Chinese Han population. Clin Sci (Lond).

[13] Zhong J, Yan Z, Liu D, Ni Y, Zhao Z, Zhu S (2006). Association of angiotensin-converting enzyme 2 gene A/G polymorphism and elevated blood pressure in Chinese patients with metabolic syndrome. J Lab Clin Med.

[14] Zhang Q, Fang W, Ma L, Wang ZD, Yang YM, Lu YQ (2018). VEGF levels in plasma in relation to metabolic control, inflammation, and microvascular complications in type-2 diabetes. Medicine (Baltimore).

[15] Holt RCL, Ralph AS, Webb NJA, Watson CJ, Clark AGB, Mathieson PW (2003). Steroid-sensitive nephrotic syndrome and vascular endothelial growth factor gene polymorphisms. Eur J Immunogenet.

[16] da Costa CCP, de Lima NS, Bento DCP, Santos RS, Reis AAS (2022). A strong association between VEGF-A rs28357093 and amyotrophic lateral sclerosis: a Brazilian genetic study. Mol Biol Rep.

[17] Fekih-Mrissa N, Mrad M, Ibrahim H, Akremi I, Sayeh A, Jaidane A (2017). Methylenetetrahydrofolate Reductase (*MTHFR*) (C677T and A1298C) polymorphisms and vascular complications in patients with type 2 diabetes. Can J Diabetes.

[18] de Lima NS, da Costa CCP, Assunção LP, Santos KF, Bento DCP, Reis AAS (2022). One‐carbon metabolism pathway genes and their non‐association with the development of amyotrophic lateral sclerosis. J Cell Biochem.

[19] Cheng J, Tao F, Liu Y, Venners SA, Hsu YH, Jiang S (2018). Associations of methylenetetrahydrofolate reductase C677T genotype with blood pressure levels in Chinese population with essential hypertension. Clin Exp Hypertens.

[20] Li A, Shi Y, Xu L, Zhang Y, Zhao H, Li Q (2017). A possible synergistic effect of MTHFR C677T polymorphism on homocysteine level variations increased risk for ischemic stroke. Medicine (Baltimore).

[21] Amer MA, Ghattas MH, Abo-Elmatty DM, Abou-El-Ela SH (2011). Influence of glutathione S-transferase polymorphisms on type-2 diabetes mellitus risk. Genet Mol Res.

[22] Little J, Higgins JPT, Ioannidis JPA, Moher D, Gagnon F, von Elm E (2009). STrengthening the REporting of genetic association studies (STREGA)- An extension of the STROBE statement. Genet Epidemiol.

[23] Lin MH, Tseng CH, Tseng CC, Huang CH, Chong CK, Tseng CP (2001). Real-time PCR for rapid genotyping of angiotensin-converting enzyme insertion/deletion polymorphism. Clin Biochem.

[24] Santos KF, Azevedo RM, Bento DCP, Santos RS, Reis AAS (2021). No association between *GSTM1* and *GSTT1* deletion polymorphisms and Amyotrophic Lateral Sclerosis: a genetic study in Brazilian patients. Meta Gene.

[25] Harries LW, Stubbins MJ, Forman D, Howard GC, Wolf CR (1997). Identification of genetic polymorphisms at the glutathione S-transferase Pi locus and association with susceptibility to bladder, testicular and prostate cancer. Carcinogenesis.

[26] Keku T, Millikan R, Worley K, Winkel S, Eaton A, Biscocho L (2002). 5,10-Methylenetetrahydrofolate reductase codon 677 and 1298 polymorphisms and colon cancer in African Americans and whites. Cancer Epidemiol Biomarkers Prev.

[27] Benjafield AV, Wang WYS, Morris BJ (2004). No association of angiotensin-converting enzyme 2 gene (ACE2) polymorphisms with essential hypertension. Am J Hypertens.

[28] Burnham KP, Anderson DR (2004). Multimodel inference: understanding AIC and BIC in model selection. Sociological Methods Res.

[29] Sen PC, Hajra M, Ghosh M, Mandal J, Bhattacharya D (2020). Emerging Technology in Modelling and Graphics. Advances in Intelligent Systems and Computing.

[B30] Mohamed S, Ashraf R, Ghanem A, Sakr M, Mohamed R (2022). Supervised machine learning techniques: A comparison. https://www.researchgate.net/publication/363870735.

[B31] López OAM, López AM, Crossa J (2022). Multivariate Statistical Machine Learning Methods for Genomic Prediction. Springer Cham.

[32] Chafai N, Hayah I, Houaga I, Badaoui B (2023). A review of machine learning models applied to genomic prediction in animal breeding. Front Genet.

[33] Nath S, Das S, Bhowmik A, Ghosh SK, Choudhury Y (2019). The GSTM1 and GSTT1 null genotypes increase the risk for type 2 diabetes mellitus and the subsequent development of diabetic complications: a meta-analysis. Curr Diabetes Rev.

[B34] Liu LS, Wang D, Tang R, Wang Q, Zheng L, Wei J (2022). Individual and combined effects of the GSTM1, GSTT1, and GSTP1 polymorphisms on type 2 diabetes mellitus risk: a systematic review and meta-analysis. Front Genet.

[35] Mergani A, Mansour AA, Askar T, Zahran RN, Mustafa AM, Mohammed MA (2016). Glutathione S-transferase Pi-Ile 105 Val polymorphism and susceptibility to T2DM in population from Turabah region of Saudi Arabia. Biochem Genet.

[36] Staels W, Heremans Y, Heimberg H, De Leu N (2019). VEGF-A and blood vessels: a beta cell perspective. Diabetologia.

[37] Younas H, Ijaz T, Choudhry N (2022). Investigation of angiotensin-1 converting enzyme 2 gene (G8790A) polymorphism in patients of type 2 diabetes mellitus with diabetic nephropathy in Pakistani population. PLoS One.

[38] Rao DK, Shaik NA, Imran A, Murthy DK, Ganti E, Chinta C (2014). Variations in the GST activity are associated with single and combinations of GST genotypes in both male and female diabetic patients. Mol Biol Rep.

[39] Cuevas S, Villar VAM, Jose PA (2019). Genetic polymorphisms associated with reactive oxygen species and blood pressure regulation. Pharmacogenomics J.

[40] McKinney BA, Reif DM, Ritchie MD, Moore JH (2006). Machine learning for detecting gene-gene interactions. Appl Bioinformatics.

