# Supplementary material for: Machine learning approaches and genetic determinants that influence the development of type 2 diabetes mellitus: a genetic association study in Brazilian patients
Source: Braz J Med Biol Res. 2024 Dec 2;57:e13957. doi: 10.1590/1414-431X2024e13957 (PMC11653484; doi:10.1590/1414-431X2024e13957)
Supplement: Supplementary file 1 [file 1414-431X-bjmbr-57-e13957-suppl.pdf]

Figure S1. Multiplex qPCR melting curves for the *GSTM1* and *GSTT1* genes. The peaks correspond to the present genotypes of *RH92600* (83°C), *GSTM1* (87°C), and *GSTT1* (89°C). In the presence of the endogenous control (*RH92600*), the absence of peaks corresponding to *GSTM1* and/or *GSTT1* allows the identification of the respective null genotypes.

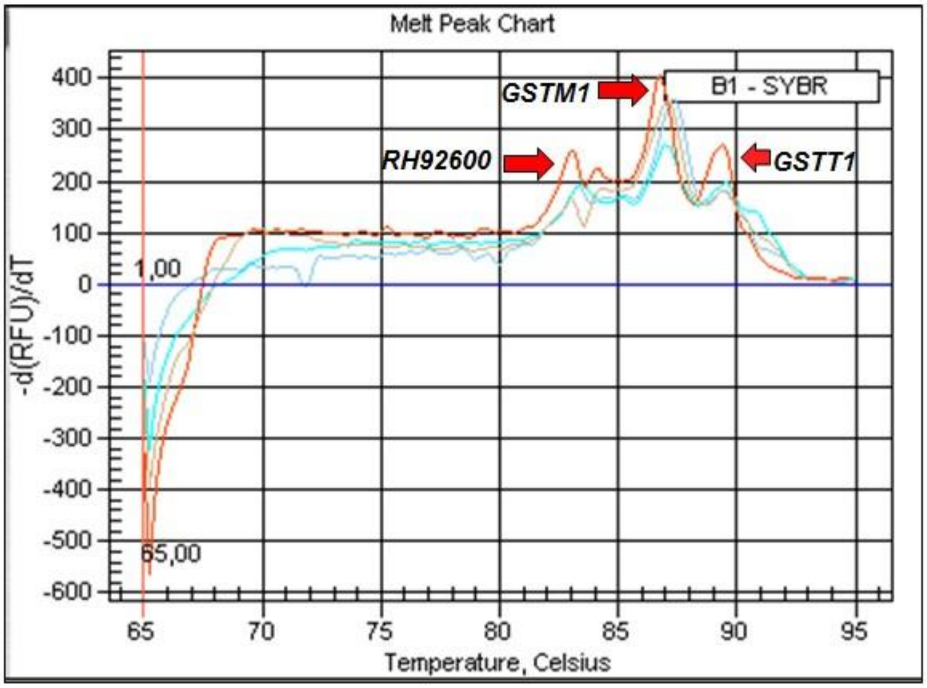

Figure S2. qPCR melting curve (SYBR Green) for the I/D polymorphism in the *ACE* gene. The peaks correspond to the insertion genotype at 73.5°C and deletion at 74°C.

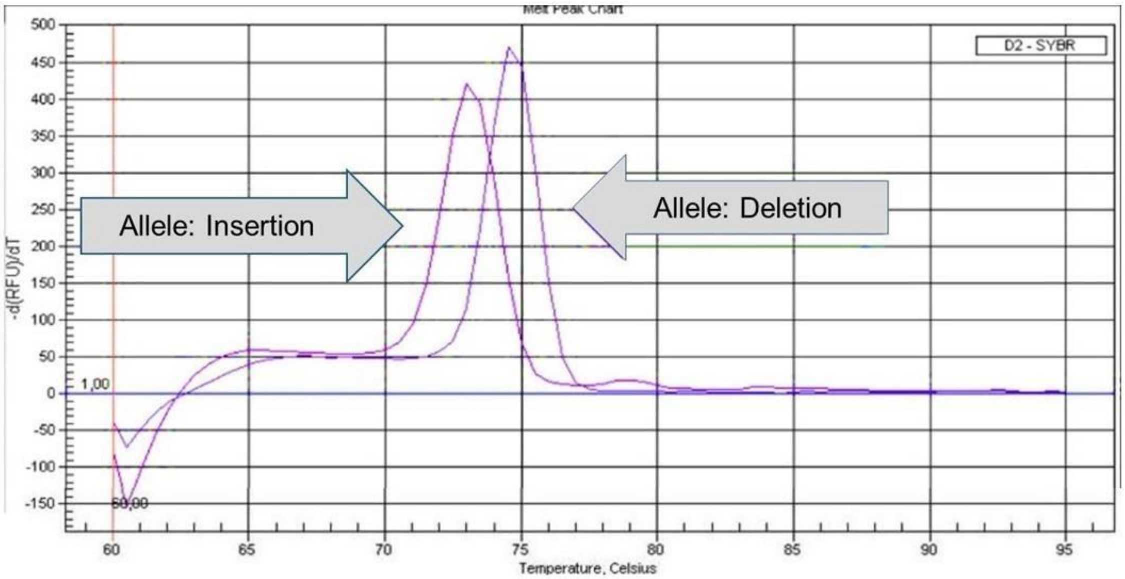

Figure S3. Polyacrylamide gel with identification of polymorphism genotypes in the *GSTP1* gene, using a 50 bp molecular marker. The wild genotype corresponds to 176 bp, the heterozygous genotype is represented by 176, 91, and 85 bp, and the homozygous mutant genotype corresponds to 91 and 85 bp.

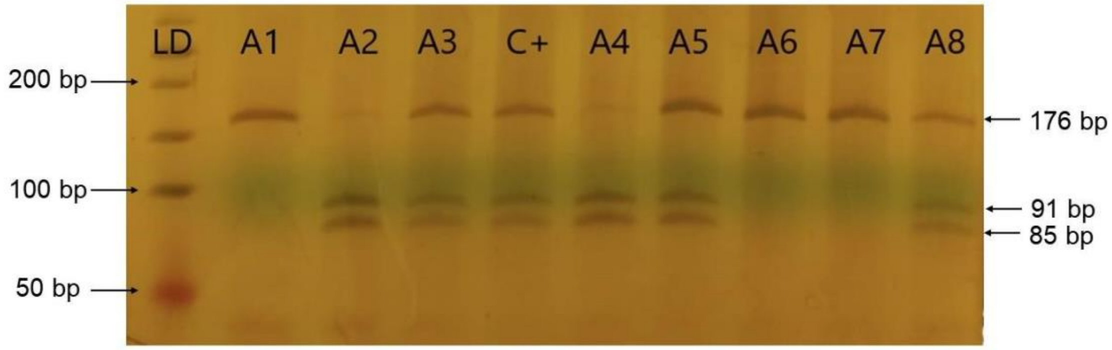

Figure S4. Polyacrylamide gel with identification of polymorphism genotypes in the *MTHFR* gene, using a 20 bp molecular marker. The wild genotype corresponds to 198 bp, the heterozygous genotype is represented by 198, 175, and 23 bp, and the homozygous mutant genotype corresponds to 175 and 23 bp.

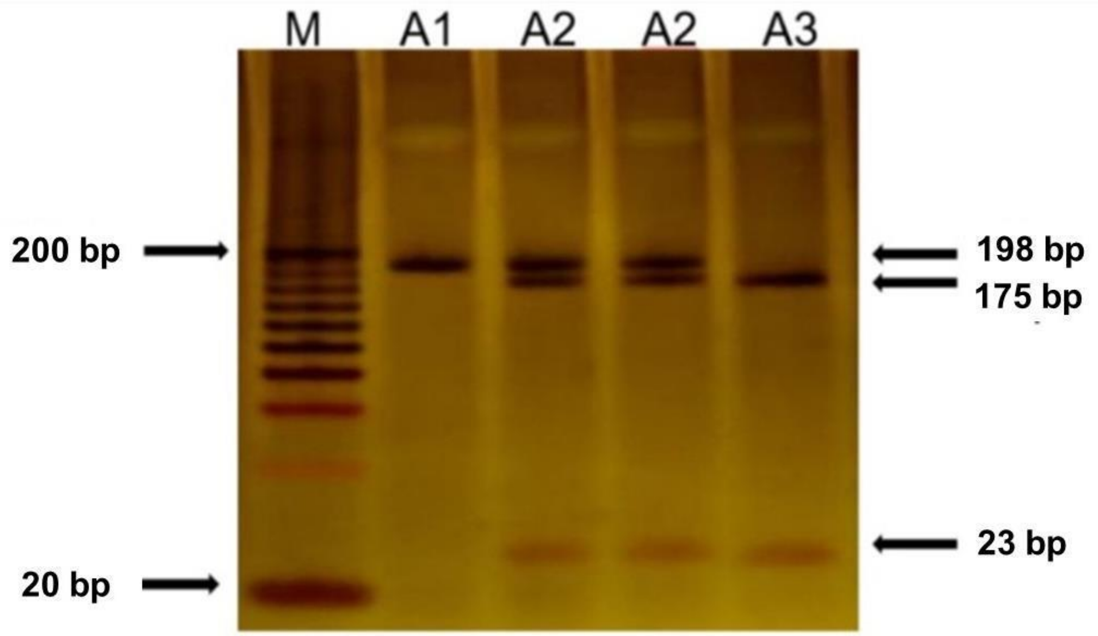

Figure S5. Polyacrylamide gel with identification of polymorphism genotypes in the *VEGF-A* gene using a 20 bp molecular marker. The wild genotype corresponds to 159 and 104 bp, the heterozygous genotype is represented by 159, 124, 104, and 35 bp, and the homozygous mutant genotype corresponds to 124, 104, and 35 bp.

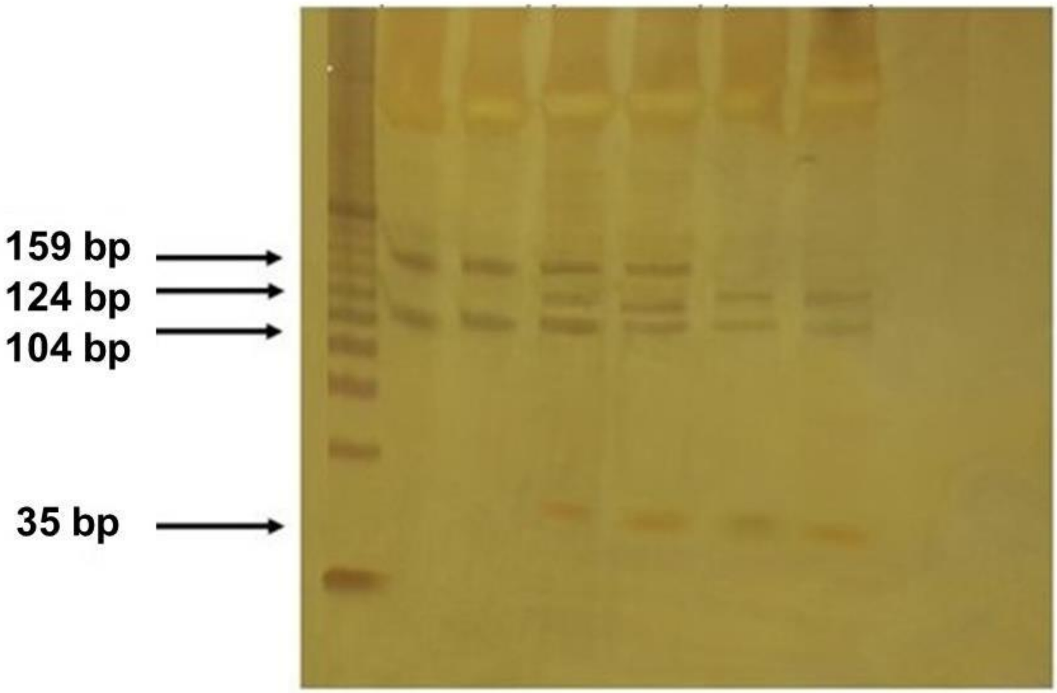

Figure S6. Polyacrylamide gel with identification of the genotypes of the polymorphism in the *ACE2* gene using a 100 bp molecular marker. The wild genotype corresponds to 466 bp, the heterozygous genotype is represented by 466, 281, and 185 bp, and the homozygous mutant genotype corresponds to 281 and 185 bp.

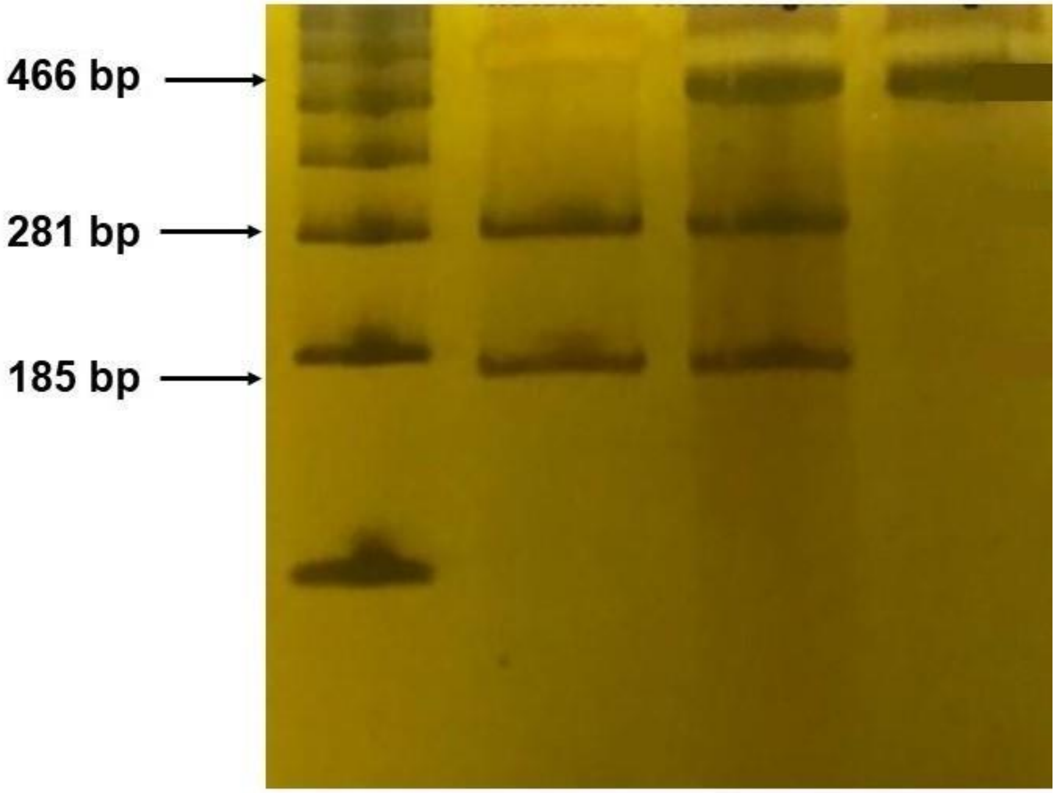

Figure S7. Permutation test with the logistic regression (LR) model for each inheritance model. **A**, codominant; **B**, dominant; **C**, recessive; **D**, overdominant. *ACE*: angiotensin converting enzyme; BMI: body mass index; Cod: codominant; Col: cholesterol; Cre: creatinine; Dom: dominant; *GSTM1*: glutathione S-transferase mu 1; *GSTP1*: glutathione S-transferase pi 1; *GSTT1*: glutathione S-transferase theta 1; HDL: high-density lipoproteins; ID: insertion/deletion; IMC: body mass index; LDL: low-density lipoprotein; *MTHFR*: methylenetetrahydrofolate reductase; Over: overdominant; PD: diastolic pressure; PS: systolic pressure; Rec: recessive; TG: triglycerides; *VEGF*: vascular endothelial growth factor; VLDL: very low-density lipoprotein.

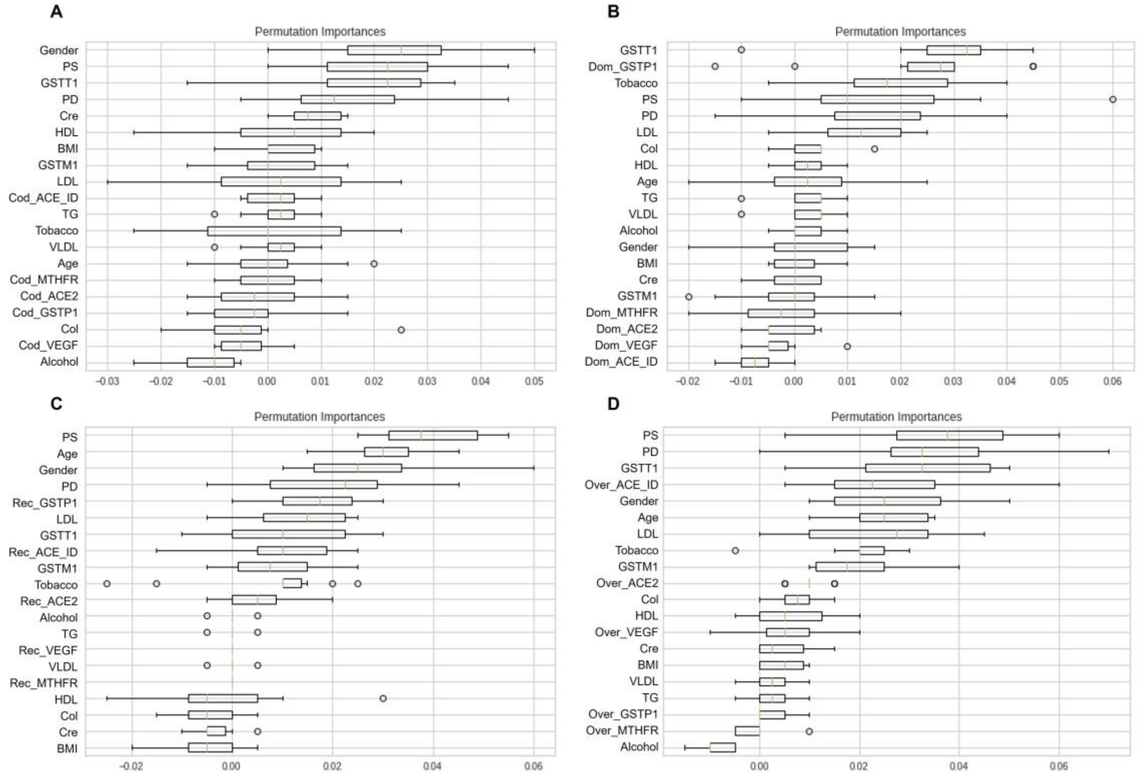

Figure S8. Permutation test with the K-nearest neighbors (KNN) model for each inheritance model. **A**, codominant; **B**, dominant; **C**, recessive; **D**, overdominant. *ACE*: angiotensin converting enzyme; BMI: body mass index; Cod: codominant; Col: cholesterol; Cre: creatinine; Dom: dominant; *GSTM1*: glutathione S-transferase mu 1; *GSTP1*: glutathione S-transferase pi 1; *GSTT1*: glutathione S-transferase theta 1; HDL: high-density lipoproteins; ID: insertion/deletion; IMC: body mass index; LDL: low-density lipoprotein; *MTHFR*: methylenetetrahydrofolate reductase; Over: overdominant; PD: diastolic pressure; PS: systolic pressure; Rec: recessive; TG: triglycerides; *VEGF*: vascular endothelial growth factor; VLDL: very low-density lipoprotein.

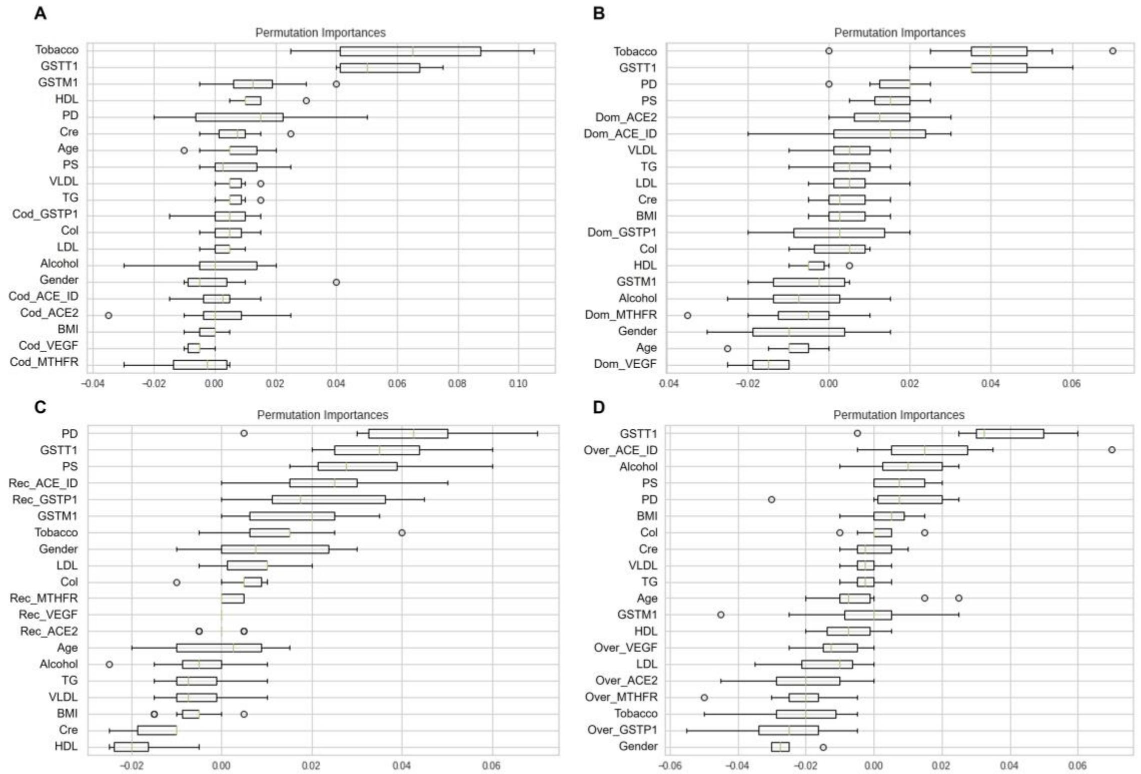

Figure S9. Permutation test with the support vector machine (SVM) model for each inheritance model. **A**, codominant; **B**, dominant; **C**, recessive; **D**, overdominant. *ACE*: angiotensin converting enzyme; BMI: body mass index; Cod: codominant; Col: cholesterol; Cre: creatinine; Dom: dominant; *GSTM1*: glutathione S-transferase mu 1; *GSTP1*: glutathione S-transferase pi 1; *GSTT1*: glutathione S-transferase theta 1; HDL: high-density lipoproteins; ID: insertion/deletion; IMC: body mass index; LDL: low-density lipoprotein; *MTHFR*: methylenetetrahydrofolate reductase; Over: overdominant; PD: diastolic pressure; PS: systolic pressure; Rec: recessive; TG: triglycerides; *VEGF*: vascular endothelial growth factor; VLDL: very low-density lipoprotein.

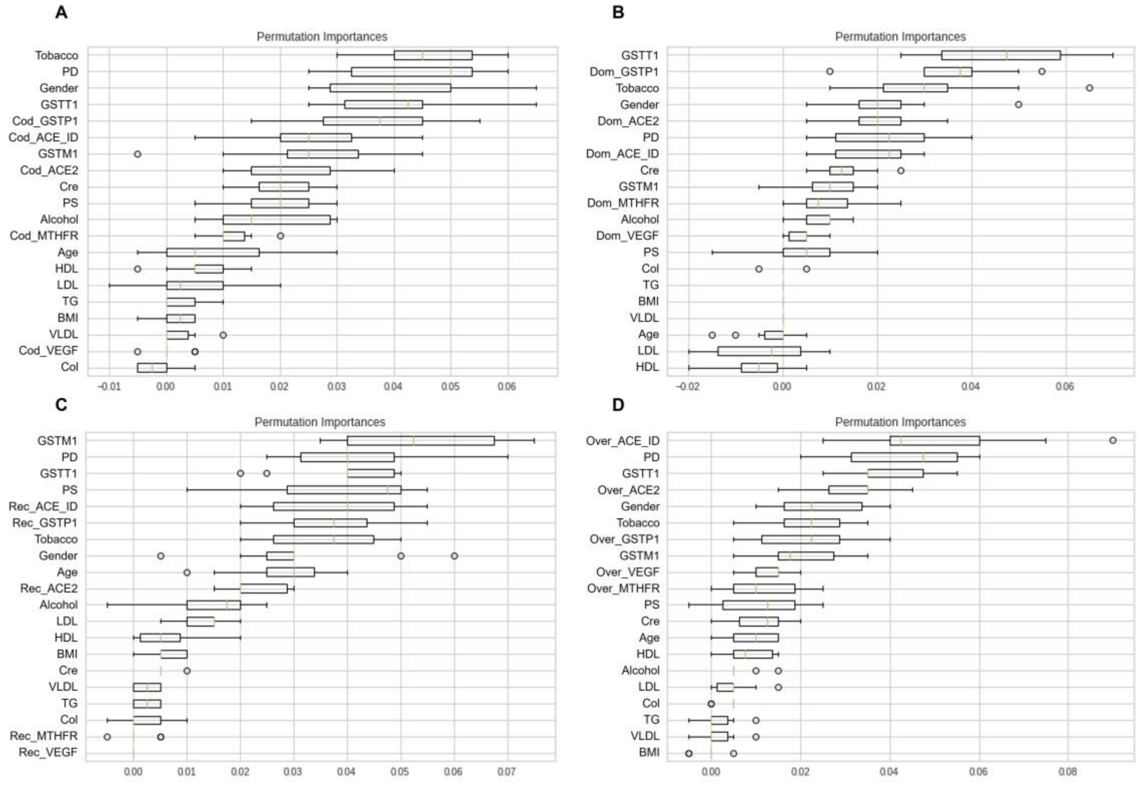

Table S1. Comparison of genotypic and allele frequencies from inheritance models and the association between diabetic and non-diabetic groups separated by sex.

			Genotypic frequency							
Gene	Model	Genotype	Male				Female			
			Control, n (%)	Case, n (%)	OR (95%CI)	P	Control, n (%)	Case, n (%)	OR (95%CI)	P
GSTT1	Dominant	P	61 (87.14)	27 (72.97)	Ref	–	85 (89.47)	58 (69.88)	Ref	–
		N	9 (12.86)	10 (27.03)	2.510 (0.91–7.01)	0.073	10 (10.53)	25 (30.12)	3.663 (1.67–8.53)	0.001*
GSTM1	Dominant	P	44 (62.86)	19 (51.35)	Ref	-	51 (53.68)	51 (61.44)	Ref	–
		N	26 (37.14)	18 (48.65)	1.603 (0.71–3.61)	0.251	44 (46.32)	32 (38.56)	0.727 (0.39–1.32)	0.297
GSTP1	Codominant	AA	14 (40.00)	16 (44.44)	Ref	–	24 (34.78)	40 (49.38)	Ref	–
		AG	18 (51.43)	14 (38.88)	0.680 (0.24–1.84)	0.451	38 (55.07)	30 (37.04)	0.473 (0.23–0.94)	0.035*
		GG	3 (8.57)	6 (16.68)	1.750 (0.38–9.53)	0.482	7 (10.15)	11 (13.58)	0.942 (0.32–2.86)	0.914
	Dominant	AA	14 (40.00)	16 (44.44)	Ref	–	24 (34.78)	40 (49.38)	Ref	–
		AG + GG	21 (60.00)	20 (55.56)	0.833 (0.32–2.14)	0.705	45 (65.22)	41 (50.62)	0.546 (0.280–1.05)	0.072
	Recessive	AA + AG	32 (91.43)	30 (83.33)	Ref	–	62 (89.85)	70 (86.42)	Ref	–
		GG	3 (8.57)	6 (16.67)	2.133 (0.51–10.82)	0.313	7 (10.15)	11 (13.58)	1.391 (0.51–3.98)	0.52
	Overdominant	AA + GG	17 (48.57)	22 (61.11)	Ref	–	31 (44.93)	51 (62.96)	Ref	–
		AG	18 (51.43)	14 (38.89)	0.601 (0.23–1.53)	0.29	38 (55.07)	30 (37.04)	0.479 (0.24–0.91)	0.027*
ACE	Codominant	II	10 (20.00)	6 (16.22)	Ref	–	27 (29.03)	28 (33.73)	Ref	–
		ID	27 (54.00)	24 (64.86)	1.481 (0.47–4.92)	0.504	52 (55.91)	38 (45.78)	0.704 (0.35–1.38)	0.309
		DD	13 (26.00)	7 (18.92)	0.897 (0.22–3.59)	0.877	14 (15.06)	17 (20.49)	1.17 (0.48–2.85)	0.726
	Dominant	II	10 (20.00)	6 (16.22)	Ref	–	27 (29.03)	28 (33.74)	Ref	–
		ID + DD	40 (80.00)	31 (83.78)	1.291 (0.43–4.15)	0.653	66 (70.97)	55 (66.26)	0.803 (0.42–1.52)	0.502
	Recessive	II + ID	37 (74.00)	30 (81.08)	Ref	–	79 (84.95)	66 (79.52)	Ref	–
		DD	13 (26.00)	7 (18.92)	0.664 (0.22–1.83)	0.439	14 (15.05)	17 (20.48)	1.453 (0.66–3.20)	0.347
	Overdominant	II + DD	23 (46.00)	13 (35.13)	Ref	–	41 (44.09)	45 (54.21)	Ref	–
		ID	27 (54.00)	24 (64.87)	1.572 (0.66–3.83)	0.31	52 (55.91)	38 (45.79)	0.665 (0.36–1.20)	0.18
ACE2	Codominant	GG	33 (64.70)	26 (70.27)	Ref	–	52 (55.32)	46 (55.42)	Ref	–
		GA	7 (13.72)	7 (18.92)	1.269 (0.38–4.15)	0.689	35 (37.23)	35 (42.17)	1.130 (0.61–2.09)	0.695
		AA	11 (21.58)	4 (10.81)	0.461 (0.11–1.52)	0.227	7 (7.45)	2 (2.41)	0.322 (0.04–1.41)	0.172
	Dominant	GG	33 (64.70)	26 (70.27)	Ref	–	52 (55.32)	46 (55.42)	Ref	–
		GA + AA	18 (35.30)	11 (29.73)	0.775 (0.30–1.90)	0.584	42 (44.68)	37 (44.58)	0.995 (0.54–1.80)	0.989
	Recessive	GG + GA	40 (78.43)	33 (89.19)	Ref	–	87 (92.55)	81 (97.59)	Ref	–
		AA	11 (21.57)	4 (10.81)	0.440 (0.11–1.42)	0.193	7 (7.45)	2 (2.41)	0.306 (0.04–1.31)	0.148
	Overdominant	GG + AA	44 (86.27)	30 (81.08)	Ref	–	59 (62.76)	48 (57.83)	Ref	–
		GA	7 (13.73)	7 (18.92)	1.466 (0.45–4.70)	0.512	35 (37.24)	35 (42.17)	1.229 (0.67–2.25)	0.503
MTHFR	Codominant	CC	22 (43.14)	18 (48.65)	Ref	–	52 (55.32)	48 (58.53)	Ref	–
		CT	27 (52.94)	19 (51.35)	0.811 (0.43–1.52)	0.73	40 (42.55)	30 (36.58)	0.812 (0.43-1.50)	0.508
		TT	2 (3.92)	0 (0.00)	0.860 (0.36–2.02)	0.992	2 (2.13)	4 (4.89)	2.166 (0.40–16.13)	0.384
	Dominant	CC	22 (43.14)	18 (48.65)	Ref	–	52 (55.32)	48 (58.53)	Ref	–
		CT + TT	29 (56.86)	19 (51.35)	0.846 (0.34–1.87)	0.608	42 (44.68)	34 (41.47)	0.876 (0.48–1.59)	0.667
	Recessive	CC + CT	49 (96.08)	37 (100.00)	Ref	–	92 (97.87)	78 (95.12)	Ref	–
		TT	2 (3.92)	0 (0.00)	0.000 (0–3.61E+108)	0.992	2 (2.13)	4 (4.88)	2.358 (0.44–17.34)	0.329
	Overdominant	CC + TT	24 (47.06)	18 (48.65)	Ref	–	54 (57.44)	52 (63.41)	Ref	–
		CT	27 (52.94)	19 (51.35)	0.938 (0.40–2.19)	0.883	40 (42.56)	30 (36.59)	0.778 (0.42–1.42)	0.42
VEGF-A	Codominant	AA	47 (92.16)	31 (86.11)	Ref	–	89 (94.68)	71 (87.65)	Ref	–
		AC	4 (7.84)	5 (13.89)	1.895 (0.46–8.18)	0.367	4 (4.25)	9 (11.11)	2.820 (087–10.75)	0.095
		CC	0 (0.00)	0 (0.00)	–	–	1 (1.07)	1 (1.24)	1.253 (0.04–32.07	0.873
	Dominant	AA	47 (92.16)	31 (86.11)	Ref	–	89 (94.68)	71 (87.65)	Ref	–
		AC + CC	4 (7.84)	5 (13.89)	1.895 (0.46–8.18)	0.367	5 (5.32)	10 (12.35)	2.507 (0.85–8.35)	0.107
	Recessive	AA + AC	51 (100.00)	36 (100.00)	Ref	–	93 (98.93)	80 (98.76)	Ref	–
		CC	0 (0.00)	0 (0.00)	1.162 (0.04–29.71)	0.916	1 (1.07)	1 (1.24)	1.162 (0.04–29.71)	0.916
	Overdominant	AA + CC	47 (92.16)	31 (86.11)	Ref	–	90 (95.74)	72 (88.89)	Ref	–
		AC	4 (7.84)	5 (13.89)	1.895 (0.46–8.18)	3.67	4 (4.26)	9 (11.11)	2.812 (0.87–10.72)	0.096

Gene	Model	Alleles	Allele frequency							
			Male				Female			
			Control, n (%)	Case, n (%)	OR (95%CI)	P	Control, n (%)	Case, n (%)	OR (95%CI)	P
GSTP1	–	A	46 (65.71)	46 (63.89)	–	0.861	86 (62.32)	110 (67.90)	–	0.331
		G	24 (34.29)	26 (36.11)			52 (37.68)	52 (32.10)		
ACE	–	I	47 (47.00)	36 (48.65)	–	0.878	106 (56.99)	94 (56.62)	–	1
		D	53 (53.00)	38 (51.35)			80 (43.01)	72 (43.38)		
ACE2	–	G	73 (71.57)	59 (79.73)	–	0.29	139 (73.93)	127 (76.50)	–	0.622
		A	29 (28.43)	15 (20.27)			49 (26.07)	39 (23.50)		
MTHFR	–	C	71 (69.61)	55 (74.32)	–	0.611	144 (76.59)	126 (76.83)	–	1
		T	31 (30.39)	19 (25.68)			44 (23.41)	38 (23.17)		
VEGF-A	–	A	98 (96.08)	67 (93.06)	–	0.491	182 (96.81)	151 (93.21)	–	0.138
		C	4 (3.92)	5 (6.94)			6 (3.19)	11 (6.79)		

Logistic regression. *P<0.05. A: adenine; C: cytosine; D: deletion; G: guanine; I: insertion; N: Null; OR: odds ratio; P: present; Ref: reference; T: thymine.

Table S2. Screening for selection of polymorphisms to be used in the combined analysis, the statistically significant polymorphisms were selected for each inheritance model.

Gene	Codominant	Dominant	Recessive	Overdominant
GSTM1	0.51	0.51	0.51	0.51
GSTT1	0.00	0.00	0.00	0.00
GSTP1	0.04	0.09	0.43	0.02
ACE	0.96	0.90	1.00	0.93
ACE2	0.10	0.83	0.07	0.54
MTHFR	0.75	1.00	0.77	0.82
VEGF-A	0.25	0.18	1.00	0.15

P<0.10 (Pearson's chi-squared test).
